# Quantifying promoter activity during the developmental cycle of *Chlamydia trachomatis*

**DOI:** 10.1038/srep27244

**Published:** 2016-06-06

**Authors:** Yanguang Cong, Leiqiong Gao, Yan Zhang, Yuqi Xian, Ziyu Hua, Hiba Elaasar, Li Shen

**Affiliations:** 1 Department of Microbiology, Immunology, and Parasitology, Louisiana State University Health Sciences Center, New Orleans, LA 70112, USA; 2 Department of Microbiology, Third Military Medical University, Chongqing, China, 400038; 3 Department of Neonatology, Children’s Hospital of Chongqing Medical University, Ministry of Education Key Laboratory of Child Development and Disorders, Chongqing Key Laboratory of Pediatrics, Chongqing, China, 400014

## Abstract

*Chlamydia trachomatis* is an important human pathogen that undergoes a characteristic development cycle correlating with stage-specific gene expression profiles. Taking advantage of recent developments in the genetic transformation in *C. trachomatis*, we constructed a versatile green fluorescent protein (GFP) reporter system to study the development-dependent function of *C. trachomatis* promoters in an attempt to elucidate the mechanism that controls *C. trachomatis* adaptability. We validated the use of the GFP reporter system by visualizing the activity of an early *euo* gene promoter. Additionally, we uncovered a new *ompA* promoter, which we named P3, utilizing the GFP reporter system combined with 5′ rapid amplification of cDNA ends (RACE), *in vitro* transcription assays, real-time quantitative RT-PCR (RT-qPCR), and flow cytometry. Mutagenesis of the P3 region verifies that P3 is a new class of *C. trachomatis* σ^66^-dependent promoter, which requires an extended −10 TGn motif for transcription. These results corroborate complex developmentally controlled *ompA* expression in *C. trachomatis.* The exploitation of genetically labeled *C. trachomatis* organisms with P3-driven GFP allows for the observation of changes in *ompA* expression in response to developmental signals. The results of this study could be used to complement previous findings and to advance understanding of *C. trachomatis* genetic expression.

*Chlamydia trachomatis* is a Gram-negative obligate intracellular bacterium that is responsible for considerable morbidity and socioeconomic burden worldwide^1^,^2^. *C. trachomatis* serovars A-C produce trachoma, a leading cause of blindness in developing countries. Serovars D–K cause the most common sexually transmitted bacterial infections. Serovars L1–L3 result in lymphogranuloma venereum (LGV), a chronic infection of the lymphatic system. Over 70% of women with *C. trachomatis* genital tract infections are asymptomatic and, if left untreated, severe sequelae may include pelvic inflammatory disease and infertility. Unfortunately, no vaccine against *C. trachomatis* is currently available. A better understanding of bacterial adaptation and pathogenesis is imperative for developing effective control strategies against the pathogen.

Bacterial cells of *Chlamydia* spp. grow solely in membrane-bound vacuoles known as inclusions within the host cell. A hallmark of the chlamydial developmental cycle is the reversible transition between two functionally divergent forms: infectious elementary bodies (EBs) and replicating reticulate bodies (RBs)^3^,^4^. During the developmental cycle, the expression of *C. trachomatis* genes is tightly regulated. Three temporal classes of *C. trachomatis* genes, early (EB-to-RB germination), middle (RB multiplication), and late (terminal RB-to-EB differentiation) have been revealed^5^,^6^,^7^. Bacterial gene transcription relies on RNA polymerase (RNAP) holoenzyme, which consists of a core enzyme and a σ factor. The σ factor confers the ability to initiate promoter-specific transcription on the enzyme^8^. *C. trachomatis* utilizes three σ factors, σ^66^, σ^54^, and σ^28^, in addition to other regulators, to control its transcription^9^. Many *C. trachomatis* housekeeping genes are transcribed by σ^66^ and a set of late-genes are regulated by σ^28^, whereas the target genes of σ^54^ remain unclear^10^,^11^,^12^,^13^,^14^. Both *C. trachomatis* σ^66^ and σ^28^ belong to the *E. coli* σ^70^ family, containing four functionally conserved domains, regions 1 to 4^15^. Despite a significantly condensed genome and limited gene regulation toolbox compared to many other bacteria, *C. trachomatis* can change its regulatory networks of gene expression rapidly in response to internal metabolic changes and external stimuli for its adaptation and survival^7^,^16^,^17^. A significant hurdle to a detailed understanding of the underlying mechanisms of gene regulation is in part the lack, until recently, of genetic systems to assess the properties of temporal promoters. Whereas the study of *C. trachomatis* promoters has been often conducted *in vitro* and in heterogeneous “*in vivo*” systems, such approaches are unable to elucidate information critical to the understanding of the *C. trachomatis* developmental cycle.

Recent advances in genetic transformation^18^ enable the visualization of *C. trachomatis* growth and the study of its protein localization using shuttle plasmid-encoded fluorescent proteins *in situ*^19^,^20^,^21^,^22^. Here, we expanded the repertoire of these powerful tools to investigate the development-dependent action of promoters in *C. trachomatis*. We constructed a transcription vector, which contains a *C. trachomatis* promoter linked to green fluorescent protein (GFP) gene. We validated its use by evaluating promoter activity of the *C. trachomatis euo* (early upstream open reading frame) gene encoding a broad DNA binding protein^23^. We also uncovered a new *ompA* promoter, P3, using the reporter assays combined with 5′ rapid amplification of cDNA ends (RACE), real-time RT-qPCR, flow cytometry, and an *in vitro* transcription assay. *C. trachomatis ompA* encodes the major outer membrane protein (MOMP), which consists of 60% of total outer membrane proteins^24^ and serves as a general porin and cytoadhesin^25^ vital to the infection process. Our results contribute to a deeper understanding of gene regulatory mechanisms by defining transcription signals of *ompA* promoters during the *C. trachomatis* development cycle. The use of the GFP reporter system offers an efficient tool to assess development-dependent changes in gene regulation in the intact cells. The knowledge acquired from this study can be used to complement previous findings and to advance studies on regulation of *C. trachomatis* genes.

## Results and Discussion

### A shuttle plasmid-based GFP reporter driven by an early *C. trachomatis* promoter

**Figure 1:**
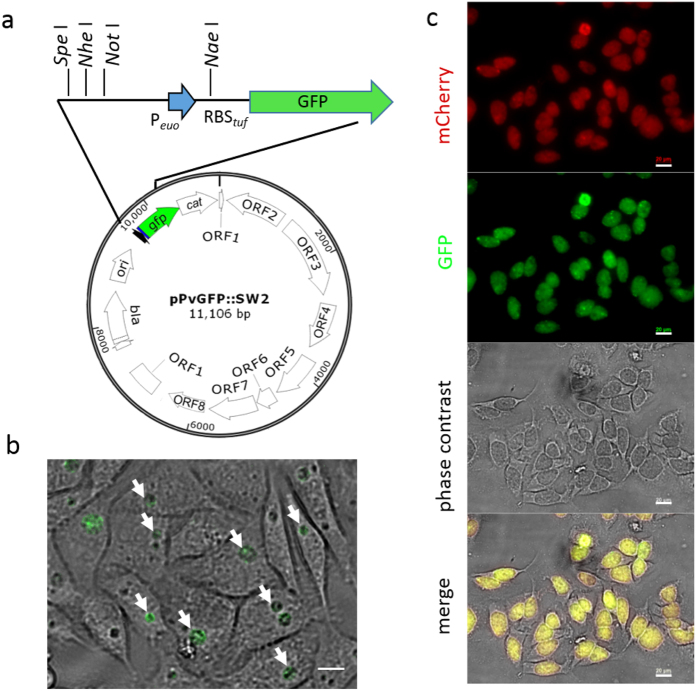
A promoter from the *euo* gene drives GFP expression in *C. trachomatis*. (**a**) Map of transcription reporter vector pPvGFP::SW2. A core promoter from the *C. trachomatis euo* gene was cloned upstream of the *tuf* RBS region and the *gfp* gene. The unique restriction sites are indicated. Bar = 10 μm. (**b**) Appearance of living L2/pPvGFP::SW2 infected HeLa cells at 16 h pi. GFP-expressing inclusions (green) are shown with arrows. (**c**) Appearance of living L2/pBOMB-P_*euo*_ infected cells at 40 h pi. P_*tet*_-controlled mCherry (red) was induced by adding αTC (20ng/ml) immediately after infection. Bar = 20 μm.

To assess promoter activity in a desirable *in situ* setting, we constructed a transcriptional reporter vector, pPvGFP::SW2 (Fig. 1a) using the backbone of the *Escherichia coli* and *C. trachomatis* shuttle plasmid, pGFP::SW2^18^. Plasmid pPvGFP::SW2 contained (i) a core promoter region (−38 to + 6 relative to the transcription start site (TSS) +1) from the *C. trachomatis euo* gene^26^ (P_*euo*_) that is flanked with a multiple cloning site (MCS) to facilitate cloning and to validate the reporter vector, (ii) a 34 bps region containing a ribosome binding site (RBS) from the *C. trachomatis tuf* gene^11^, which codes for translation elongation factor EF-Tu at the 5′ end of the *gfp* gene. Previous studies showed that *tuf* RBS functioned in both *C. trachomatis* and in *E. coli*^10^,^11^, ensuring translation of reporter genes. The plasmid also contained the *bla* gene encoding β-lactamases for positive selection in *C. trachomatis*. The plasmid pPvGFP::SW2 was transformed into *C. trachomatis* L2/25667R, a naturally occurring plasmid-free strain. After four rounds of selection with ampicillin, GFP-expressing inclusions were observed by fluorescent microscopy. A clone of the transformed strain, named L2/pPvGFP::SW2, was used to infect HeLa cells. GFP-expressing inclusions appeared at 16 hours post-infection (h pi) (Fig. 1b). The levels of GFP increased proportionately to the expansion of *C. trachomatis* inclusions and accumulation of organisms. The control, transformed with the promoter-less plasmid, pPLGFP::SW2, did not express GFP. This data indicates that GFP expression is specifically driven by P_*euo*_.

To facilitate GFP quantitation in *C. trachomatis* using an internal protein control, a DNA fragment containing MCS-P_*euo*_-*tuf* RBS from pPvGFP::SW2 was subcloned into pBOMB4-tet-mCherry^20^ (hereafter called pBOMBm), which contained the fluorescent protein mCherry gene driven by a tetracycline controlled promoter (P_*tet*_). The resultant plasmid, pBOMB-P_*euo*_, was transformed into L2/25667R cells to generate L2/pBOMB-P_*euo*_. Infection of L2/pBOMB-P_*euo*_ in HeLa cells formed GFP-expressing inclusions at 16 h pi and beyond, a phenotype similar to that of L2/pPvGFP::SW2. P_*tet*_-driven mCherry expression was induced in the presence of anhydrotetracycline hydrochloride (αTC) (Fig. 1c). Thus, the level of mCherry expressed from the same plasmid provided a control to normalize the levels of GFP expression. Previous studies indicated that the *euo* gene mRNA was moderately expressed as early as 2 h pi from *Chlamydia* spp.^6^,^26^. The failure to detect P_*euo*_-driven GFP expression prior to 16 h pi by fluorescence microscopy was perhaps due to the small inclusion size and low bacterial accumulation. It is also possible that the core P_*euo*_ region used in the construct is intrinsically weak and (an) additional DNA element(s) upstream or downstream may be required for its optimal expression in *C. trachomatis*. Nevertheless, our results indicate that GFP can be produced under the control of P_*euo*_ and the *tuf* translation machinery ribosome complex.

### Prediction of *ompA* P3

**Figure 2:**
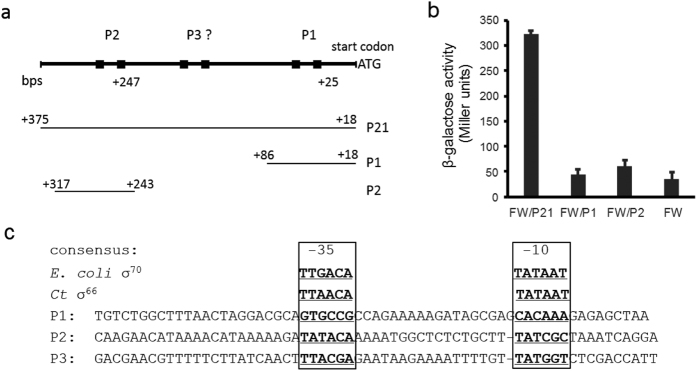
Studying *C. trachomatis ompA* promoter activity in *E. coli*. (**a**) Promoter-*lacZ* constructions. The organization of the regulatory region upstream of the *ompA* coding region (top) and DNA fragments used to create the *lacZ* reporter strains are shown. Positions relative to the translation start codon ATG of *ompA* are indicated. (**b**) Results of β-gal activity. *E. coli* strains, FW/P21, FW/P2, and FW/P1, were harvested at the exponential phase and subjected to β-gal assays. Strain FW (vector only) was used as a control. Data are presented as mean ± SD from a representative experiment of three independent experiments. (**c**) Base sequences of putative *ompA* promoters used in this work. Putative −35/−10 hexamers are bolded. The *E. coli* σ^70^ or *C. trachomatis* σ^66^ consensus recognition sequences are listed at the top.

*C. trachomatis* surface-exposed MOMP, encoded by the *ompA* gene, is a critical component that directly mediates pathogen-host interactions^25^. Developmentally regulated *ompA* transcription has been a topic of extensive study^12^,^14^,^27^,^28^. Although *ompA* is expressed as multiple mRNAs, only the P1-derived short transcript and the P2-derived long transcript have been studied in detail^12^,^14^,^29^. To quantify the effects of DNA regions on *ompA* promoter activity, we created a set of *lacZ* transcriptional fusions in *E. coli* based on Whipple’s system^30^. The regions carrying the promoters (either combined P2 and P1, or P1 or P2 alone) were cloned into the *lacZ* expression vector, pFW11, and the promoter-*lacZ* fusion was relocated to an F′ episome in the recipient cells as detailed in the Materials and Methods section. The resultant strains, designated FW/P21, FW/P1, or FW/P2 (Fig. 2a), were subjected to β-galactosidase (β-gal) assays to measure the activity of *ompA* promoters. We observed a 6-fold increase in β-gal activity in FW/P21 cells compared to the promoter-less control (Fig. 2b). However, no β-gal levels exceeding that of the promoter-less control were produced in FW/P2 or FW/P1. These results are comparable to previous studies using multi-copy plasmid-encoded chloramphenicol acetyltransferase (CAT) as a reporter in *E. coli*^12^. Although the activity of P2 has been authenticated in *C. trachomatis*, the role of P1 is still under debate^12^,^29^. It is unsurprising that P2 is not active in *E. coli* because P2 has a GC-rich −10 hexamer of TATCGC, which is not recognized by *E. coli* σ^70^ *in vitro*^29^. The high levels of β-gal observed in strain FW/P21 could therefore not be explained by the lack of β-gal activity in strains FW/P1 and FW/P2. We suspected that an additional promoter recognized by *E. coli* transcription machinery must be present between the P2 and P1 regions.

We next searched for the putative promoter (s) from the 607 bp intergenic sequences (IGSs) upstream of the *ompA* of *C. trachomatis* strain L2/434/Bu *in silico* using a dynamic program-based MotifSearch tool^10^. A two-block-motif, TTAACA (−35)-n_16–19_-TATAAT (−10), was used for *C. trachomatis* σ^66^ recognition sequences^31^ and motif TAAAGTTT (−35)-n_10–14_-GTTGACAA (−10) was used for σ^28^ recognition sequences^10^. Up to 3 base-pair mismatches (a total 6 bases) were allowed in the −35 hexamer or −10 hexamer. These predictions resulted in the identification of the known *ompA* P2 region and a region containing the sequence TTACGA-n_17_-TATGGT, which we named P3 (Fig. 2c). The same P3 region was predicted using a position weight matrix-based program called Footy^32^. P1 was excluded, as it contained mismatches in five of six positions in the putative −35 hexamer and in three of six positions in the −10 hexamer. No σ^28^ recognition sequences were found. An alignment of *Chlamydia* spp. IGSs upstream of *ompA* available in the NCBI database shows a minor variation in P3 and P2 regions (Table S1). This suggests that these regions may have a conserved role in *ompA* expression in *Chlamydia* spp. There are more variations in the *ompA* P1 region in terms of the length of the “spacer” separating the putative −35/−10 hexamers, ranging from 18 to 26 bps. *Chlamydia* spp. σ^66^-dependent promoters typically have a 16–18 bp spacer^11^,^31^, resembling those of *E. coli* σ^70^, which also senses the promoter spacing and preferentially recognizes a spacer with an average of 17 bps^33^.

### Validation of P3 function in *C. trachomatis*

**Figure 3:**
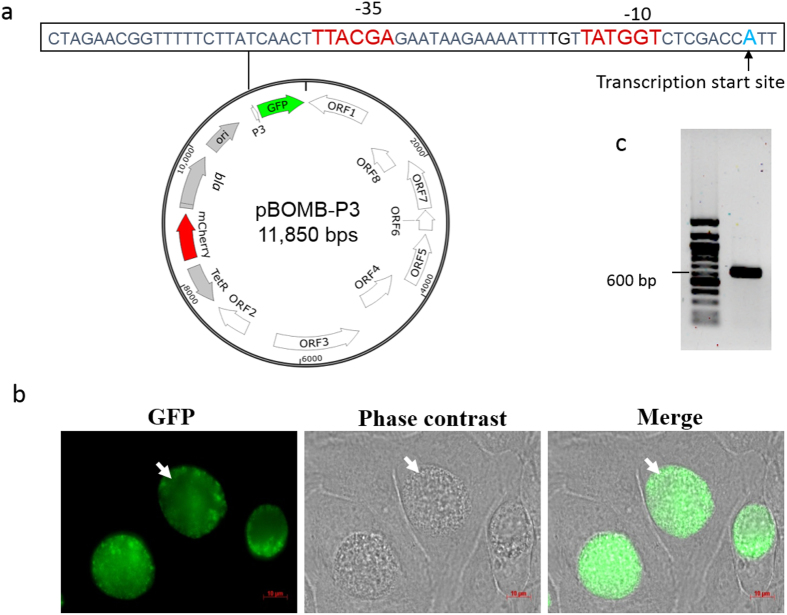
Determining P3 activity in *C. trachomatis*. (**a**) Map of pBOMB-P3. Sequences of P3 region are indicated. (**b**) Appearance of living L2/pBOMB-P3 infected HeLa cells at 30 h pi. GFP-expressing inclusions are marked with white arrows. (**c**) The *ompA* P3 transcriptional start site (TSS) was identified by 5′-RACE assays. The PCR products next to the DNA ladder on the agarose gel were subjected to sequencing, and the TSS of P3 appears to be “A” (in blue font), marked with an arrow. The putative −35 and −10 hexamers are indicated in red font.

To directly determine whether P3 functions in *C. trachomatis,* the 63 bp P3 region was cloned into pPvGFP::SW2 and pBOMB-P_*euo*_, in place of P_*euo*_, generating pP3GFP::SW2 and pBOMB-P3 (Fig. 3a), respectively. These plasmids were each transformed into the L2/25667R cells, resulting in L2/pP3GFP::SW2 and L2/pBOMB-P3. After three rounds of ampicillin selection, GFP-expressing inclusions were observed (Fig. 3b). Inclusion morphology in both strains carrying pP3GFP::SW2 and pBOMB-P3 appeared to be normal and not significantly different. Strain L2/pBOMB-P3 was chosen for further characterization.

Next, 5′ RACE assays were performed to determine the transcription start site (TSS) of P3-derived mRNA. Initially, total RNA isolated from the strain L2/434/Bu infected HeLa cells harvested at 18 h pi were used. Our attempt to detect the 5′ end of P3 mRNA was unsuccessful, although a site corresponding to the reported P1 mRNA^14^,^27^ was noted. We hypothesized that if the undetectable 5′ end of the P3 transcript is the result of low steady-state levels of P3 mRNA, the use of the *C. trachomatis tuf* RBS region should improve the stability of P3 mRNA in the reporter system. To test this hypothesis, P3 transcript was determined using a 5′ RACE assay with total RNA isolated from L2/pBomB-P3 infected HeLa cells harvested at 18 h pi. The DNA sequencing data showed that P3 transcript originated with an adenine located at 39 bp upstream from the *gfp* start codon (Fig. 3c). Based on the TSS at position +1, the sequences of TTACGA and TATGGT are putative −35 and −10 hexamers of P3. Yuan *et al*.^28^. have previously reported a similar site in *C. psiticci* Mn Cal 10 and *C. psiticci* GPIC (now *C. caviae* according to current chlamydiae taxonomy)^34^. We conclude that P3, lying between the previous reported P2 and P1, indeed functions actively in *C. trachomatis.*

### Defining the core P3 promoter

**Figure 4:**
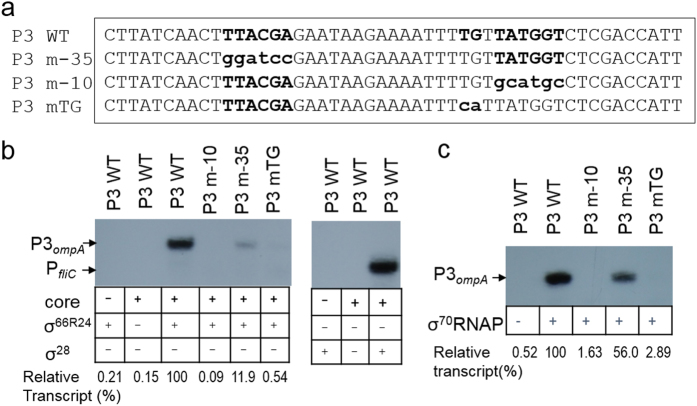
Identifying the determinants of *ompA* P3 region. (**a**) Sequences of P3 and its derivatives tested in this study. (**b**) Autoradiogram of a denaturing acrylamide gel showing the transcript products of *in vitro* transcription assays with P3 or its derivatives. σ^66R24^ RNAP holoenzyme (left panel) or σ^28^ RNAP holoenzyme (right panel) were used. (**c**) An autoradiogram of a denaturing acrylamide gel showing *ompA* P3 transcripts produced in the transcription assay. *E. coli* σ^70^ RNAP holoenzyme was used. The amounts of transcripts were determined by densitometry using ImageJ^49^ and are shown relative to WT P3, which is set at 100%, as indicated at the bottom of (**b,c**). Note: P3 is transcribed by both σ^66R24^ RNAP and σ^70^ RNAP, but not σ^28^ RNAP.

To precisely define the relevant sequence features needed for the recognition of P3, we investigated how specific mutations of P3 could affect transcription *in vitro*. Plasmids containing wild-type (WT) or mutated P3 regions (Fig. 4a) and a control promoter of the *E. coli fliC* (P_*fliC*_)^35^ were used as templates. A functional RNAP holoenzyme was reconstituted with core enzyme from *E. coli* and a hybrid *C. trachomatis* σ factor, σ^66R24^, in which σ^66^ regions 2 to 4 (amino acid residues 315–571) were translationally fused to the region 1 of *E. coli* σ^70^ (amino acid residues 1–372). The use of this holoenzyme allows for the study of σ^66^-dependent promoter activity that relies on the function of σ^66^ regions 2 to 4. *E. coli* σ^70^ or *C. trachomatis* σ^28^ were used as controls. In the presence of σ^66R24^- or σ^70^-RNAP holoenzyme, transcripts from WT P3 were evident (Fig. 4b,c). In contrast, no P3–derived transcript was observed in the presence of σ^28^. These data indicate that P3 is specifically recognized by *C. trachomatis* σ^66^ and its homolog *E coli* σ^70^, consistent with our β-gal reporter assay data in *E. coli* (Fig. 2). Substitution of the TATGGT (−10 hexamer) with GCATGC abolished P3 transcription activity when either σ^66R24^RNAP or σ^70^RNAP was used, indicating the importance of the TATGGT hexamer for P3 activity. Interestingly, substitutions of the TTACGA (−35 hexamer) to GGATCC reduced 80% of P3 transcript in comparison to the WT P3 using σ^66R24^RNAP, while the use of σ^70^RNAP reduced P3 transcript only by 50%, suggesting that the TTACGA hexamer is more recognizable by *C. trachomatis* σ^66^. These results indicate that the TTACGA sequence of P3 functions as the −35 promoter element and confirm that this sequence is required for the full activity of P3. We noted that the _−16_TGt_−14_ sequences in P3 matched the consensus extended -10 TG motif recognized by σ^70^ from *E. coli*^36^,^37^. Substitution of TG with CA resulted in a significant decrease in P3 transcript levels, suggesting that the TG motif is important for P3 activity (Fig. 4). *C. trachomatis* σ^66^-dependent promoters have typically been characterized as −10/−35 promoters. Our data, for the first time, shows that P3 activity requires the −35 hexamer and an extended −10 TGn motif, presenting a new promoter class in *C. trachomatis*.

Promoters harboring the TGn motif have been identified from several microbial systems, including *E. coli*^37^, *Streptococcus pneumoniae*^38^, *Bacillus subtilis*^36^, and *Mycobacterium tuberculosis*^39^. It has been shown that the recognition of the TGn motif by the principle σ factor plays a role in the formation of an open complex during transcription initiation. Moreover, the TGn motif may compensate for non-canonical −10 hexamers or suboptimal spacer length between −10 and −35 hexamers. The TGn-promoters appear to more often have significant deviations in the –35 hexamer than typical –10/–35 promoters in *E. coli*. It seems that *ompA* P3 deviates substantially from both the consensus −10 hexamer and −35 hexamer, because of its GC-rich sequences in these regions. Like *E. coli* σ^70^, *C. trachomatis* σ^66^ region 2 binds to the promoter −10 hexamer, region 4 of σ^66^ recognizes the −35 hexamer^40^. It is likely that the TGn motif allows for extra contact points with σ^66^ regions 2.5 and 3.0, as has been observed in its *E. coli* counterpart^33^,^37^,^41^.

### Quantifying the contribution of P3 to *ompA* transcription

**Figure 5:**
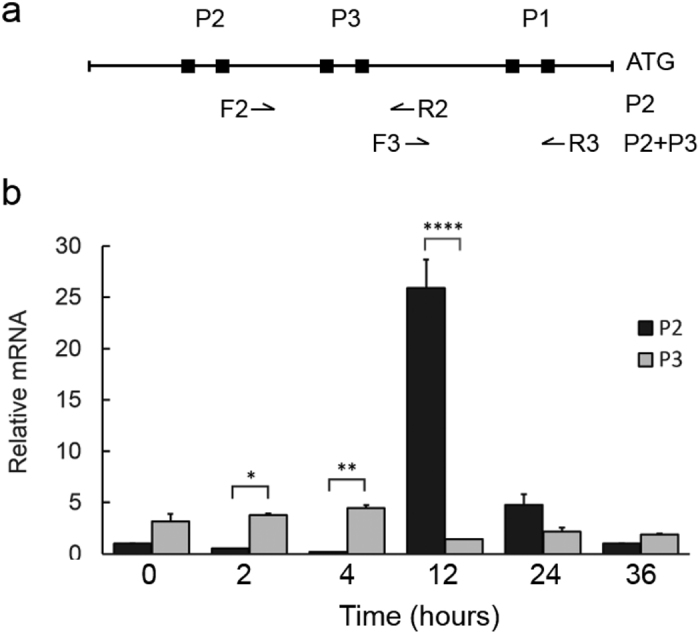
The profile of *ompA* P3 and P2 expression in chromosomal loci. (**a**) Diagram showing the location of primers designed to assess *ompA* transcripts by RT-qPCR. Amplification with the primers, F2/R2, produces P2 transcript, whereas amplification with primers, F3/R3, generates the total transcripts of P2 and P3. The individual P3 transcript is obtained by subtracting P2 transcript from both P3 and P2 transcripts. (**b**) Results of RT-qPCR with total RNA from L2/434/Bu infected HeLa cells harvested at 0, 2, 4, 12, 24, and 36 h pi. Relative amounts of transcript were obtained by normalizing the levels of P2 or P3 transcript to *C. trachomatis* genomic DNA levels, which were assessed using primers specific to the 16S rRNA gene (Table S3). The values are presented as mean ± SD from an experiment with duplicates. *P < 0.05, **P < 0.005, and ****P < 0.0001. Three independent experiments were performed.

To define the role of P3 in *ompA* transcription and to understand the potential relationship between P3 expression and the action of the well-studied P2^14^,^27^,^29^, we quantified the levels of *ompA* P2 and/or P3 mRNA in the context of chromosomal loci using real-time RT-qPCR. Total RNA from L2/434/Bu infected cells, which were sampled at 0, 2, 4, 12, 24, and 36 h pi, were analyzed (Fig. 5a). P3 transcript was detected early at a low level and continued to accumulate in abundance, exceeding that of P2 at both 2 and 4 h pi (Fig. 5b). P2 transcript rapidly increased with bacteria multiplication and peaked at 12 h pi. However, at 12 h pi, the use of P3 decreased and that of P2 drastically increased. Expression of both *ompA* P2 and P3 was significantly eliminated at 36 h pi. These data support the notion that P2 is the primary transcript during RB replication and RB-to-EB differentiation, as previously reported^27^, whereas P3 plays a role in the early stage of the development. Therefore, temporally expressed P3 and P2 coordinate *ompA* transcription during the *C. trachomatis* developmental cycle.

### Evaluating the use of P3-GFP as a reporter in *C. trachomatis* infected epithelial cells

**Figure 6:**
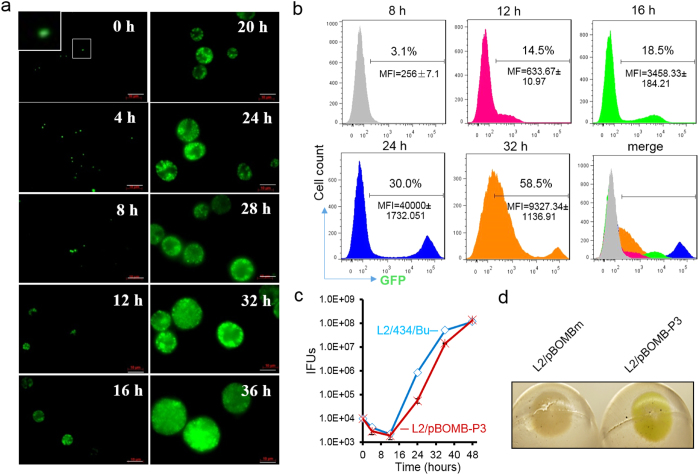
Inspecting P3-GFP levels during the course of the *C. trachomatis* infection. (**a**) Time course of GFP expression in HeLa cells visualized by fluorescent microscopy. L2/pBOMB-P3 infected HeLa cells were imaged at the times indicated. The inset highlights GFP-expressing *C. trachomatis* organisms. Bar = 10 μM. (**b**) Flow cytometric data corroborate microscopic images in (**a**), showing changes in GFP expression in *C. trachomatis* infected HeLa cells. GFP-expressing cell population is indicated as percentage. The average mean fluorescence intensity (MFI) and the standard deviation at each time is shown. (**c**) One-step growth curve of *C. trachomatis* strains L2/434/Bu and L2/pBOMB-P3. (**d**) Isolated L2/pBOMB-P3 organisms, but not L2/pBOMBm, appear to be green-colored by direct observation.

*C. trachomatis* growth is accompanied by quantitative and qualitative changes in *ompA* expression (Fig. 5). To investigate the relationship between P3-GFP level and *C. trachomatis* growth, GFP in L2/pBOMB-P3 infected HeLa cells was monitored throughout the *C. trachomatis* developmental cycle using fluorescence microscopy and flow cytometry. In parallel, a one-step growth curve was constructed to enumerate infectious EB progeny yields. We visualized GFP-expressing chlamydial organisms associated with the host cells instantly post-infection, and the levels of GFP expression increased through 36 h pi as *C. trachomatis* inclusions expanded (Fig. 6a). When quantitatively measured using flow cytometry, the GFP signal was detected instantly after infection but the signal-to-noise ratio was low, preventing meaningful quantitative measurements of cells. The distinct cell populations with *bona fide* P3-GFP expression were separate from the GFP negative cells until 8 h pi (Fig. 6b). The population of GFP-expressing cells increased from ~3.1% at 8 h pi to 30% at 24 h pi; concurrently, the mean florescence intensity (MFI) significantly increased (Fig. 6b). These increases were correlated with the rapid growth of *C. trachomatis* (Fig. 6c). Interestingly, while GFP-expressing cells increased to 58.5% at 32 h pi, there were more heterogeneous cell populations which displayed a lower average MFI with a larger deviation compared to that at 24 h pi. This could be explained by the asynchronous growth of *C. trachomatis*, which occurs when decreasing numbers of RBs continue replicating and increasing progeny EBs accumulate and exit from the host cells to initiate a new cycle of infection. The overall P3-driven GFP levels seemed to be quite stable. Thus, it is likely that the GFP signal detected at the late stage is skewed by GFP accumulation along with bacteria accretion and inclusion expansion. We observed that L2/pBOMB-P3 exhibited delayed growth pattern relative to L2/434/Bu (Fig. 6c), possibly due to the stress induced by GFP overexpression. Isolated L2/pBOMB-P3 organisms from infected cells harvested at 40 h pi appeared green-tinged, a change detectable by eye (Fig. 6d), indicating high levels of GFP associated with bacteria. Long-lived P3-GFP used in this study may be applicable for long-term bacteria cell-labeling, which allows for the imagining to track the *C. trachomatis* infection process in intact cells, e.g. invasion, multiplication, and dissemination.

### Determining the strength of P3 in *C. trachomatis*

**Figure 7:**
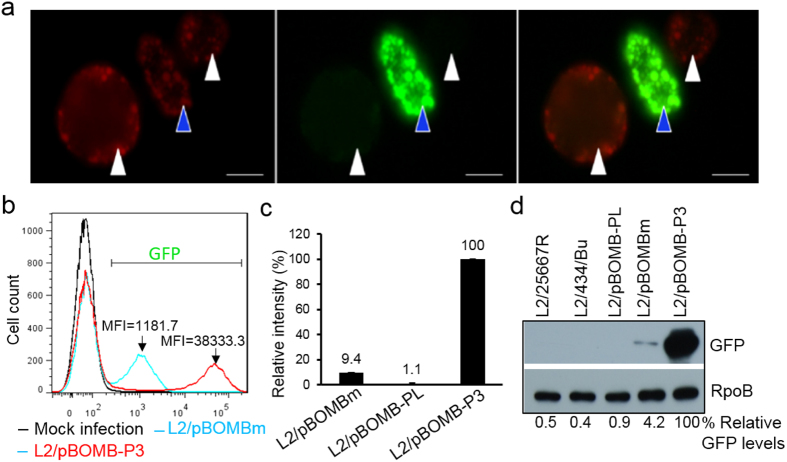
Quantitative analysis of the *ompA* P3 strength in *C. trachomatis*. (**a**) Appearance of GFP-expressing inclusions (green) upon induction of mCherry (red) expression by the addition of αTC. HeLa cells co-infected with strains L2/pBOMB-P3 (blue arrow) and L2/pBOMB-m (white arrow) were photographed at 24 h pi. Bar = 5 μm. (**b**) Flow cytometry measures of P3-GFP intensity compared to that of P_*nm*_–GFP. *C. trachomatis* infected HeLa cells were harvested at 24 h pi and subjected to flow cytometry. (**c**) Assessing GFP levels of cell-free *C. trachomatis* organisms using microspectrometry as detailed in Materials and Methods. (**d**) Immunoblotting of GFP protein. Bacteria with the same OD_600_ values (OD_600_ = 0.4) were used in the experiments. The amount of protein were determined by densitometry using ImageJ. The relative amount of GFP was obtained by normalizing the GFP intensity to the corresponding RpoB intensity and value is reported as a percentage relative to L2/pBOMB-P3, as indicated at the bottom.

To evaluate the strength of P3, we assessed the P3-driven GFP levels in L2/pBOMB-P3 compared to those driven by the *Neisseria meningitidis* promoter, P_*nm*_, in L2/pBOMBm^18^,^20^. Four different quantitative methods (microscopy, flow cytometry, microspectrometry, and immunoblotting) were used. First, to facilitate a direct comparison, an equivalent mixture of L2/pBOMB-P3 and L2/pBOMBm was used to infect HeLa cells, followed by fluorescence microscopy analysis. In the same field of vision, L2/pBOMB-P3 exhibited a brighter green fluorescence than L2/pBOMBm (Fig. 7a). In the presence of equivalent mCherry levels, which were induced by the addition of αTC, L2/pBOMB-P3 displayed higher levels of GFP signal than L2/pBOMBm. Next, cells infected with either L2/pBOMB-P3 or L2/pBOMBm with similar infectivity (~30%) were harvested at 24 h pi and subjected to flow cytometry. As determined by assessment of the MFI, the average level of P3–driven GFP expression was ~32 times stronger than P_*nm*_-driven GFP at 24 h pi, (Fig. 7b). Further, isolated *C. trachomatis* organisms from cells harvested at 24 h pi were measured for their relative fluorescence intensities (RFI) using microspectrometry. Figure 7c shows that the RFI of L2/pBOMB-P3 was ~10-fold higher than that of L2/pBOMBm. Lastly, the level of GFP protein in isolated *C. trachomatis* organisms was determined by immunoblotting. The levels of GFP in lysates of L2/pBOMB-P3 were ~24-fold higher than those of L2/pBOMBm (Fig. 7d). With the use of similar plasmid constructs, these results demonstrate that, relative to P_*nm*_, P3 is highly active in *C. trachomatis*. Furthermore, bacterial organisms expressing P3-GFP are more noticeable and easier to measure using fluorescence microscopy, flow cytometry and immunoblotting, rather than microspectrometry.

### *OmpA* P3 displays weak transcription activity in *E. coli*

**Figure 8:**
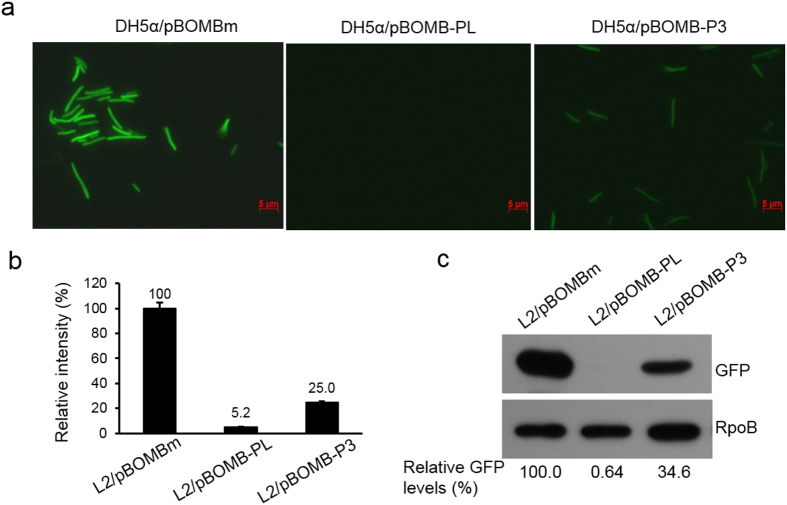
The *OmpA* P3 is weakly active in *E. coli*. (**a**) Visualizing GFP expression in *E. coli* DH5α cells harboring pBOMBm, promoter-less pBOMB-PL, and pBOMB-P3 by fluorescence microscopy. Fixed *E. coli* cells harvested from LB cultures grown for 3 hours were used. Bar = 5 μm. (**b**) Assessment of GFP intensity of *E. coli* cells using microspectrometry as detailed in the Materials and Methods section. (**c**) Quantification of GFP by immunoblotting. Bacterial lysates were used for immunoblotting with antibodies to GFP or RpoB which was used as a loading control for protein amounts. The relative amount of GFP was obtained by normalizing the GFP intensity to the corresponding RpoB intensity and value is reported as a percentage relative to L2/pBOMB-P3, as indicated at the bottom.

Our next step was to determine whether P3 activity varies with the *E. coli* σ^70^ paradigm. This information may provide new insight into the diversity of bacterial gene transcription and also may provide an explanation for the previous detection of P3 in *E. coli* systems. To this end, *E. coli* strains carrying pBOMB-P3, pBOMBm, and promoter-less pBOMB-PL were harvested at the exponential phase and their GFP expression was measured using fluorescence microscopy, microspectrometry, and immunoblotting. A significant increase in GFP expression in *E. coli* cells was observed as the bacterial culture grew (Fig. 8a), confirming that P3 was recognized by *E. coli* RNAP. This data is consistent with the earlier observations as shown in Figs 2 and 4b. Unlike in *C. trachomatis*, where P3 is highly expressed, P3-driven GFP expression was much lower than P_*nm*_-driven GFP in *E. coli* (Fig. 8b,c). The weak activity of P3 in *E. coli* is not surprising, as perfect P3 function may be achieved only in the context of a regulatory network unique to *C. trachomatis*; different regulatory mechanisms exist in *E. coli*.

Given the high similarity between *C. trachomatis* σ^66^ and *E. coli* σ^70^ in their regions 1 to 4, the use of heterogeneous *E. coli* systems has provided useful information to understand some questions regarding chlamydial promoter recognition as described both previously and in this study. However, these heterogeneous systems do not allow for the expression of all *C. trachomatis* genes, such as *ompA* P2^29^. P3 functions weakly in *E. coli* and appears to rely on the availability of endogenous conditions for optimal expression in *C. trachomatis*. Such variety might be attributed to the non-conserved regions between *C. trachomatis* σ^66^ and *E. coli* σ^70^, including the area between regions 1 and 2, as well as the regions at the N- and C-termini of σ factors^42^,^43^. Differences between *C. trachomatis* σ^28^ and *E. coli* σ^28^ have been studied previously^10^,^44^. Additionally, physiological signals within the host cells (such as immunity and metabolic capacity) that *C. trachomatis* encounters affect bacterial gene expression. Therefore, it is necessary to validate the promoters that were predicted or identified by the heterologous systems in *C. trachomatis*. A major advantage of the transcription reporter shuttle plasmid with an MCS and *tuf* RBS upstream of GFP is to allow a preferred promoter to be inserted conveniently so that promoter functioning can be studied by assessing the levels of GFP expression. Promoters detected in *C. trachomatis* could be directly compared to those in *E. coli*, thus providing an opportunity to examine the extent of the differences in their activity.

## Conclusion

This study resulted in three significant conclusions. First, the *ompA* regulatory region is complex and exhibits novel features. In particular, *ompA* transcription is exactly derived from both mid-cycle expressed P2 and the newly identified, early-expressed P3. Second, P3 represents a new class of *C. trachomatis* promoter, which requires a −35 DNA element and an extended −10 TGn motif for its transcription. Third, expression of *ompA* P3-GFP, but not P_*euo*_-GFP, in living cells produces a unique, robust signal. These results provide a basis for a deeper understanding of novel characteristics of the gene regulation in *C. trachomatis*.

Tandemly arranged promoters have been reported to involve developmentally regulated *C. trachomatis* gene expression, including *ompA*^27^,^28^ and *tuf*^11^. To complement the earlier finding that P2 plays a primary role in *ompA* synthesis, we have shown that P3 also contributes to *ompA* transcription (Figs 2, 3, 4, 5). Previous studies have indicated the detection of steady-state levels of *ompA* transcript mid-cycle^6^,^7^. However, the levels of *ompA* transcripts often do not fully reflect the relative activities of the promoters because the fate of each transcript may be varied and is affected by various processes. The switch in utilization of P3 and/or P2 is evidently a regulatory mechanism of *ompA* transcription. The manner in which P3 and P2 activity is coordinated or mutually influenced can be affected by multiple factors. A 9-bp inverted repeat adjacent to the −35 hexamer of the P3 region may play a role in this process. Mathews and Stephens^12^ have proposed that this repeat, resembling an operator in DNA structure, might be a target of a yet not-identified protein repressor. Alternatively, a stable stem-loop structure that resembles a rho-independent terminator capable of stopping P2 transcription could be formed by this region. Additionally, DNA supercoiling has been reported to modify *C. trachomatis* transcription, including *ompA* P2^45^. Only a subset of early genes can be affected by DNA supercoiling^46^. In general, plasmids are supercoiled to a higher degree than chromosomal DNA. We found similar strong activity of P3 regardless of its plasmid and chromosomal locations, suggesting that supercoiling is less likely to be a main factor involved in P3 expression. Moreover, posttranscriptional regulation of *ompA* mRNA may occur during development. Transcript derived from P3 seems less stable than that of P2 by mRNA structural prediction. Despite its role in early transcription, P3 mRNA might limit its activity through quick turnover when RBs rapidly replicate and convert to EBs. We cannot exclude the possibility that P1 signal detected is the processed product of longer transcripts^14^, perhaps from P3 and/or P2. It is worth noting that the utility of *tuf* RBS in mediating the translation of *gfp* may be mechanistically different from, and possibly more efficient than, the native *ompA* RBS. Because of the apparent conservation of the P3 and P2 regions, their importance in *Chlamydia* spp. adaptation to changing environments can be predicted. The proposed P1 remains unproven, but phylogenetic analysis suggest that P1 is less likely to be the conserved regulatory mechanism of *ompA* in *Chlamydia* spp.

Our findings underscore the role of new promoter element, the extended −10 TGn, in *C. trachomatis* transcription. By sequence inspection, early promoters in *C. trachomatis*, P_*euo*_ and the promoter from *C. trachomatis groES*^13^, also contain potential extended −10 TGn motifs. It is possible that such a motif represents a signature element for early promoter-RNAP recognition and is affected differently from the typical −35/−10 promoters by a transcription factor. For example, we have found that anti-σ factor, CT663 (or Scc4)^40^, strongly inhibited transcription from the −35/−10 promoters but was less effective in suppressing transcription from an extended −10 promoter lacking a −35 element. Despite the limited number of transcription factors in *C. trachomatis*, the arrangements of promoter modules may be largely diverse and greatly contribute to developmentally regulated gene expression profiles.

The consistency of the different quantitative methods employed show that robust P3-GFP signal is present throughout the developmental cycle. These properties make P3-GFP valuable for long-term bacterial cell-labeling to facilitate the visualization and tracking of infectious process in live cells. Coupled with flow cytometry and microscopy, this GFP-reporter assay may also be applicable in probing *ompA* changes induced by various insults at both population and single cell level. Adverse growth conditions, such as exposure to IFNγ or antibiotics, cause *C. trachomatis ompA* downregulation and the formation of an “altered persistent form”^17^,^47^. With regard to this, low levels of GFP expression driven by a weak promoter, such as P_*euo*_, could be challenging to interpret. At the same time, the use of long-lived GFP may be limited, as it is incapable of fully reflecting the highly dynamic process of gene expression. An improved short-lived GFP reporter may be more suitable for studying the transient mRNA dynamics in *C. trachomatis*. All together, the results obtained and approaches used in this study will allow for the design of future studies in defining promoter signatures, relationships between promoter structure, RNAP recognition, and transcriptional activity in *C. trachomatis*.

## Materials and Methods

### Cell cultures and bacteria growth

HeLa 229 cells (human cervical epithelial carcinoma cells; ATCC CCL-2) were cultured in Dulbecco’s Modified Eagle Medium (DMEM) supplemented with 1 mM glutamic acid, 10% fetal bovine serum and 20 μg/ml gentamycin (DMEM-10) at 37 °C in an incubator supplied with 5% CO_2_. HeLa cells were infected with *C. trachomatis* as previously described^17^. To construct a one-step growth curve, cells infected at an MOI of 1 were lysed and cultured on fresh HeLa cells at 0, 4, 12, 24, 32, and 48 h pi. Infectious EB progeny was then evaluated by enumeration of inclusion forming units (IFUs) in 1 ml. *E. coli* DH5α was used as the host for cloning. A methylation deficient *E. coli* strain (ER2925, New England Biolabs) was used to prepare the plasmids for *C. trachomatis* transformation. The *E. coli* cells were grown in Luria-Bertani (LB) broth or agar plates containing the appropriate antibiotics.

### Molecular cloning

Plasmids, primers, and oligonucleotides used in this study are listed in Table S2 and Table S3, respectively. Primers and oligonucleotides were synthesized by Integrated DNA Technologies. To construct pPvGFP::SW2 (Fig. 1a), two separated PCR fragments were obtained with primers, Euo_tuf priF/ORF2 priF or euoPriR/ORF1priF, using pGFP::SW2^18^ (a gift form Dr. Ina Clarke, University of Southampton, UK) as template. These PCR fragments were mixed, annealed, and used as templates for subsequent PCR using primers ORF2 priF/ORF1priF. The *Bam* HI-cut PCR fragment was then inserted into the sites of *Bam* HI in pGFP::SW2. To create pP3GFP::SW2, the two annealed complementary oligonucleotides consisting of *ompA* P3 were cloned into pPvGFP::SW2 at the *Spe* I/*Nae* I sites. The *Puv* I/*Nco* I fragment of the pP3GFP::SW2 was cloned into the *Pvu* I/*Nco* I sites in pBOMB4-tet-mCherry (a gift form Ted Hackstadt, Rocky Mountain Lab, NIH) to yield pBOMB-P3 (Fig. 3a). To create a promoter-free plasmid, pPvGFP::SW2 was digested by *Spe* I/*Nae* I to remove P_*euo*_. After Kenow fragment treatment, the DNA was self-ligated to create pPLGFP::SW2. The *Puv* I/*Nco* I fragment was subcloned into pBOMB to create pBOMB-PL. The plasmids used for the *in vitro* transcription assay were derived from pP_*flic*_^35^. Two annealed complementary oligonucleotides consisting of WT or mutated P3 were inserted into pP_*flic*_ at the *Xba* I/*EcoR* V sites to yield pP3WT-P_*fliC*_, pP3m10-P_*fliC*_, pP3mTG-P_*fliC*_ or pP3m35-P_*fliC*_. The expression plasmid, pLN-σ^66R24^, containing genes encoding *C. trachomatis* σ^66^ regions 2–4 and *E. coli* σ^70^ region 1, was derived from pLNH-12. The PCR fragment consisting of region 1 of *E. coli* σ^70^ was amplified with primers s70priF/Sig7066Rg24BLo. The PCR fragment containing σ^66^ regions 2–4 was amplified with primers sig7066Rg24CUp/S66prRBam. These DNA fragments were annealed and used as a template for subsequent PCR using primers EC70priF/S66prRBam. The resultant PCR fragments were digested with *Nco* I/*Bam* HI and were then inserted into the *Nco* I/*BamH* I sites of pLN-7066 to generate pLN-σ^66R24^, which encodes a fusion σ^66R24^ protein. The identities of all constructs were confirmed by PCR and DNA sequencing.

### Generating *ompA* promoter-*lac Z* fusion in *E. coli*

A DNA fragment containing the *ompA* P1 and P2 regions (+375 to +18 relative to the translation start codon ATG of *ompA*) was amplified by PCR with primers *ompA*priF/P1priR using the genome of *C. trachomatis* serovar D as a template. The *EcoR* I/*Sal* I digested PCR fragment was then inserted into the *Eco* RI/*Sal* I sites of pFW11 to yield pFW/P21. Two annealed oligonucleotides containing P2 (+317 to +243 relative to the *ompA* start codon) or P1 (+87 to +18 relative to the *ompA* start codon) were inserted into pFW11 at *Eco* RI/*Sal* I sites, resulting in pFW/P2 or pFW/P1 (Fig. 2a). These constructs were introduced into *E. coli* CSH100 for homologous recombination of the *ompA* promoter::*lacZ* onto an F’ episome as previously described^30^. Further mating with *E. coli* strain FW102 was performed to finalize the construction of the reporter strains, designated as FW/P21, FW/P2, and FW/P1, respectively. The identities of all constructs and strains were confirmed by PCR and DNA sequencing.

### Transformation of *C. trachomatis*

*C. trachomatis* was transformed with the shuttle plasmids according to the method described^18^ with minor modifications. Briefly, plasmid-free *C. trachomatis* L2/25667R EBs (1 × 10^7^) were mixed with 7 μg of plasmid DNA in 100 μl of CaCl_2_ buffer (5 mM Tris HCl, pH7.4, 100 mM CaCl_2_) and incubated at room temperature for 30 minutes. Freshly trypsinized HeLa cells (6 × 10^6^) resuspended in 200 μl CaCl_2_ buffer were added to the plasmid/EB mixture and incubated at 37 °C for an additional 20 min. Aliquots of this mixture were then added to a 6-well plate with 1.0 ml of pre-warmed medium in each well. After culturing in DMEM-10 without antibiotics at 37 °C for 24 h, cells were incubated in the presence of ampicillin (5 μg/ml) and cycloheximide (20 μg/ml) for an additional 24 hours. The infected cells were harvested and lysed by vortexing with glass beads. The cell debris was removed by spinning at 233 *g* for 10 min. The *Chlamydia*-containing supernatant was collected and added onto a HeLa monolayer in a T175 flask. After incubation at 37 °C for 1 hour with gentle shaking, the infected cells were cultured in DMEM-10 containing ampicillin and cycloheximide for 48 hours. The *C. trachomatis* organisms were isolated by 30% renografin density gradient purification to enrich infectious EBs. For the first passage, a HeLa monolayer in a 6-well plate was inoculated with EBs, followed by centrifugation to enhance infection. Passages were continued 2–3 times, until inclusions positive for green fluorescence were observed. To isolate a single clone of *C. trachomatis* transformant, GFP-expressing cells were lysed in sterile dH_2_O, serially diluted, and inoculated onto a HeLa cell monolayer in a 96-well plate. At 24 h pi, the plate was examined by fluorescence microscopy. The culture wells containing only a single inclusion were marked and continued to be cultured until subsequent harvest for *C. trachomatis* large scale amplification.

### Phase contrast and fluorescence microscopy

HeLa cell monolayers grown in a glass culture chamber (NUNC) or 24-well culture plate were infected with *C. trachomatis* to achieve ~30% infection. At various times post infection, the culture wells were observed and photographed with an inverted fluorescence microscope (Zeiss Axio Observer D1). Images were processed using AxioVision software version 4.8.

### Microspectrometry

Isolated chlamydial organisms from infected cells harvested at 36 h pi were washed with PBS, diluted to OD_600_ = 0.4, and subjected to fluorescence intensity analysis using a Synergymx microplate reader (BioTek) with excitation wavelengths of 48 nm and 528 nm. A volume of 100 μl of bacterial cell dilutions was added into each well of a 96-well plate. The following formula was used to determine relative fluorescence intensity (RFI): RFI = (bacterial fluorescence intensity–background)/bacterial OD_600_. The overnight cultures of *E. coli* DH5α carrying GFP expression plasmids were diluted in fresh LB (1:100) and grown at 37 °C. *E. coli* cells grown at log phase (4 hours after inoculation) were collected (0.5 ml) by centrifugation and resuspended in the same volume of PBS. The fluorescence intensity of *E.coli* was obtained in the same manner as described above for *C. trachomatis*.

### Flow cytometry

*C. trachomatis* infected HeLa 229 cells were detached by trypsinization at different times as indicated in the results and fixed with 2% paraformaldehyde at room temperature for 20 minutes. Cells were then detected in a FACSVantage flow cytometer (BD Biosciences) using the FL-1 (green) channel. Flow cytometry data was recorded for at least 3 × 10^4^ cells per sample. Mock infection cells were used as a blank. Data were analyzed using FlowJo software Version 7.6 (TreeStar Inc) for both the percentage of GFP-expressing cell populations and mean fluorescence intensity.

### Immunoblotting analysis

Chlamydial organisms were lysed with 2 × SDS loading buffer, separated on 4–20% SDS-PAGE, and transferred onto a PVDF membrane for immunoblotting. The membrane was incubated with a polyclonal anti-GFP antibody (Pierce) or a monoclonal antibody to bacterial RNAP β subunit (RpoB) (Neoclone), followed by incubation with horseradish peroxidase-conjugated goat anti-mouse IgG. RpoB was used as a protein loading control. The protein bands were visualized by an enhanced chemiluminescence kit (Pierce).

### 5′ Rapid amplification of cDNA ends (RACE)

*C. trachomatis*-infected cells with ~90% infectivity were harvested at 18 hours. Total RNA was isolated using TRIzol reagent (Invitrogen). The transcription start site for P3 in *C. trachomatis* was probed with the rapid amplification of the cDNA ends (5′-RACE) using the FirstChoice ^®^ RLM-RACE Kit version 2.0 (Life Technologies) following the manufacturer’s protocol. Briefly, 10 μg of total RNA were treated with calf intestine alkaline phosphatase to remove free 5′-phosphates. The RNA was then treated with tobacco acid pyrophosphatase to remove the cap structure from full-length mRNA, leaving a 5′-monophosphate. An RNA adapter oligonucleotide was then ligated to the RNA with 5′-phosphate using T4 RNA ligase. The 5′ end of the *gfp* transcript, which is initiated by P3, was then amplified with random-primer reverse transcription and nested PCR using *gfp* specific primers with the provided primers in the kit. PCR products were then inserted into pUC19 (Promega, Madison, WI) for DNA sequencing.

#### Real-time reverse transcription quantitative PCR (RT-qPCR)

*C. trachomatis* infected HeLa cells were harvested at indicated times post infection. Genomic DNA was isolated from cells using the DNeasy^®^ Blood & Tissue Kit (Qiagen). RNA was isolated from an equivalent number of cells using the Direct-zol™ RNA Kit (Zymo). DNase treatment was performed to remove residual DNA. A total of 1 μg of RNA was reverse transcribed into cDNA and the genes of interests were amplified using the VeriQuest Fast SYBR Green qPCR (USB) with appropriate primers (Table S2). The PCR cycle conditions were as follows: 50 °C for 2 min., 95 °C for 5 min., 95 °C for 3 sec., and 60 °C for 30 sec. The latter two steps were repeated for 35 to 45 cycles, and fluorescence was detected at the end of each cycle. Transcripts were normalized to *C. trachomatis* genomic DNA levels, which were quantified by qPCR with primers specific to the 16S rRNA gene^48^ (Table S3). The 2-ddCt method was used to obtain relative transcript levels.

### Recombinant protein purification, RNAP and the *in vitro* transcription assays

Recombinant *C. trachomatis* σ^66R24^ was expressed in *E. coli* Rosetta^TM^ (DE3) pLysS cells harboring pLN-σ^66R24^. Cells were grown in LB broth at 37 °C to *A*_600_ = 0.8, and then protein expression was induced by the addition of isopropyl-1-thio-β-d-galactopyranoside (IPTG) to a final concentration of 0.5 mM. Protein purification was performed using anion exchange chromatography (Source Q, GE Healthcare), followed by gel filtration purification as previously described^40^. Recombinant *C. trachomatis* σ^28^ was purified as previously described^35^. To make a functional RNAP holoenzyme, the purified σ^66R24^ (2.0 μg) was mixed with 1 unit of *E. coli* RNAP core (Epicentre Technologies) and incubated on ice for 30 min. The σ^28^-RNAP holoenzyme was reconstituted using *E. coli* core and purified σ^28^. *E. coli* σ^70^ RNAP holoenzyme was purchased from USB. An *in vitro* transcription assay was performed as described previously^10^,^40^, in a 10 μl reaction containing 1 μl 10 × RNA Pol reaction buffer (New England Biolab), 1 μl RNAP holoenzyme, 1 μg of plasmid template, 400 μM ATP, 400 μM UTP, 1.2 μM CTP, 0.2 μM [α-^32^P]CTP, 100 μM 3′-O-methylguanosine 5′-triphosphate (GE HealthCare), 2 mM DTT, and 20 U RNase inhibitor (USB). The reaction was incubated for 15 min at 37 °C and terminated by adding loading buffer. The transcripts were separated by electrophoresis with 6% polyacrylamide/8 M urea gel and visualized by autoradiography.

### Statistical analyses

Data analyses were performed using GraphPad PRISM software. Statistical significance was determined by two-way analysis of variance (ANOVA). Values of *P < 0.05 were considered statistically significant.

## Additional Information

**How to cite this article**: Cong, Y. *et al*. Quantifying promoter activity during the developmental cycle of *Chlamydia trachomatis. Sci. Rep.* **6**, 27244; doi: 10.1038/srep27244 (2016).

## Supplementary Material

Supplementary Information
