# Supplementary material for: Quantifying promoter activity during the developmental cycle of Chlamydia trachomatis
Source: Sci Rep. 2016 Jun 6;6:27244. doi: 10.1038/srep27244 (PMC4893696; doi:10.1038/srep27244)
Supplement: Supplementary Information [file srep27244-s1.doc]

**Supplemental information**

**Quantifying promoter activity during the developmental cycle of *Chlamydia trachomatis***

Yanguang Cong, Leiqiong Gao, Yan Zhang, Yuqi Xian, Ziyu Hua, Hiba Elaasar, and Li Shen

**Table S1. Promoter regions of *ompA* in various strains of *Chlamydia* spp.**

Strains ^a^ sequence alignment ^b^

CT A/Har-13 CCAAGAACATAAAAACATAAAAAGA**TATACA**AAAATGGCTC-TCTGCTT**TATACA**TAA--ATCAGGAGG---CGCTTAAGGGCTTCTTCCTGGGACGAAC

CT B/Jali20-OT CCAAGAACATAAAAACATAAAAAGA**TATACA**AAAATGGCTC-TCTGCTT**TATACA**TAA--ATCAGGAGG---CGCTTAAGGGCTTCTTCCTGGGACGAAC

CT C/TW-3 ACAAGAATATAAAAACATAAAAAGA**TATACA**AAAATGGCTC-TCTGCTT**TATACA**TAA--ATCAGGAAG---CGCTTAAAGGCTTCTTCCTGGGACGAAC

CT D/14-96 CCAAGAACATAAAAACATAAAAAGA**TATACA**AAAATGGCTC-TCTGCTT**TATACA**TAA--ATCAGGAGG---CGCTTAAGGGCTTCTTCCTGGGACGAAC

CT E/SW3 CCAAGAACATAAAAACATAAAAAGA**TATACA**AAAATGGCTC-TCTGCTT**TATACA**TAA--ATCAGGAGG---CGCTTAAGGGCTTCTTCCTGGGACGAAC

CT F/SW4 CCAAGAACATAAAAACATAAAAAGA**TATACA**AAAATGGCTC-TCTGCTT**TATACA**TAA--ATCAGGAGG---CGCTTAAGGGCTTCTTCCTGGGACGAAC

CT G/9301 CCAAGAACATAAAAACATAAAAAGA**TATACA**AAAATGGCTC-TCTGCTT**TATACA**TAA--ATCAGGAGG---CGCTTAAGGGCTTCTTCCTGGGACGAAC

CT H CCAAGAACATAAAAACATAAAAAGA**TATACA**AAAATGGCTC-TCTGCTT**TATACA**TAA--ATCAGGAAG---CGCTTAAAGGCTTCTTCCTGGGACGAAC

CT Ia/20-97 CCAAGAACATAAAAACATAAAAAGA**TATACA**AAAATGGCTC-TCTGCTT**TATACA**TAA--ATCAGGAGG---CGCTTAAGGGCTTCTTCCTGGGACGAAC

CT J/31-98 CCAAGAACATAAAAACATAAAAAGA**TATACA**AAAATGGCTC-TCTGCTT**TATACA**TAA--ATCAGGAAG---CGCTTAAAGGCTTCTTCCTGGGACGAAC

CT K/SotonK1 CCAAGAACATAAAAACATAAAAAGA**TATACA**AAAATGGCTC-TCTGCTT**TATACA**TAA--ATCAGGAGG---CGCTTAAGGGCTTCTTCCTGGGACGAAC

CT L1/115 CCAAGAACATAAAAACATAAAAAGA**TATACA**AAAATGGCTC-TCTGCTT**TATACA**TAA--ATCAGGAGG---CGCTTAAGGGCTTCTTCCTGGGACGAAC

CT L2/434/Bu CCAAGAACATAAAA-CATAAAAAGA**TATACA**AAAATGGCTC-TCTGCTT**TATCGC**TAA--ATCAGGAGG---CGCTTAAGGGCTTCTTCCTGGGACGAAC

CT L2b/795 CCAAGAACATAAAAACATAAAAAGA**TATACA**AAAATGGCTC-TCTGCTT**TATACA**TAA--ATCAGGAGG---CGCTTAAGGGCTTCTTCCTGGGACGAAC

CT L2c CCAAGAACATAAAA-CATAAAAAGA**TATACA**AAAATGGCTC-TCTGCTT**TATCGC**TAA--ATCAGGAGG---CGCTTAAGGGCTTCTTCCTGGGACGAAC

CT L3/404/LN CCAAGAACATAAAAACATAAAAAGA**TATACA**AAAATGGCTC-TCTGCTT**TATACA**TAA--ATCAGGAGG---CGCTTAAGGGCTTCTTCCTGGGACGAAC

CAB S26-3 CTAAGTGGCATAAGAAATAAAAATG**TGTACA**AAAATCTAATAGCTCTCT**TATTAG**CAAGTATAAGGAGTTATACATTGAAA-TCTGTGCCTGAAAACAGT

CAV 10DC88 CTAAACAGCATAAGAAATAAAAA-G**TGTACA**AAAATCCCGCGGTTCGCT**TATTAG**CAAGCATAAGGAATGATTGTTTAAAAACCCATGCCGGAAAATAGT

CCA GPIC CTAAGTAGCATAAGAAATAAAAGCG**TGGACA**AAAATCTATTAGCTAACT**TATTAG**CACGTATAAGGAGTTAGTGCTTGAAG-CCTATGCCTGGAAACAGC

CFE Fe/C-56 CTAAGTGGCATAAGAAATAAAAGTG**TGTACA**AAAATCTATTAGCCCATT**TATTAG**CGTATTTAAGGAGTTATGGCTTGAAA-CCTATGCCTGAAAACTGT

CG JXC CTAAGCAGCATAAGAAACAAAAAAG**TATACA**AAAATTCCTAGTCTCACT**TATTAG**CAAGCATAAGGAATGATTGCTTAAAA-CTTATGCCAGAAAATAGT

CM MoPn/Nigg TCTGCAAAAACCAAGCATAAAAAGA**TATACA**AAAATGGAGC-TCTACTT**TATACA**TAG--GTCAGAAGA---CGCTTAAAAGCTTCTTCCTGAGACAGAC

CPE E58 GAAAAACTCTTCTCAAGAAAAAACA**TATACA**AAAAAACCTCTTCCCTCC**TATAGA**AC--TCTTAGAGGTCCTTGTTTGAAAACACTGTCTGAATTCACAC

CPN TW-183 TAAATTATATATATAATGAAAAGAA**TATACA**AAAAAGCTATAGCTTTCC**TATAGC**TCA-TAACAGAAGTTCTTGGTTGAAATGTGCGGCTAAAAACACTT

CPS 6BC CTAAGTGGCATAAGAAATAAAAATGTGTACAAAAATCTGATAGCTCTTT**TATTAG**CAAGTATAAGGAGTTATACATTGAAA-TCTATGCCTGAAAACAGT

P2 -35 P2 -10

CT A/Har-13 GTTT-TTCTTATCAACT**TTACGA**GAATAAGAAAATTT**TG**T**TATGGT**CTCGAGCATTGAACGACATGTTCTCGATTAAGGCTGCTTT-TACTTGCAAGACA

CT B/Jali20-OT GTTT-TTCTTATCAACT**TTACGA**GAATAAGAAAATTT**TG**T**TATGGT**CTCGAGCATTGAACGACATGTTCTCGATTAAGGCTGCTTT-TACTTGCAAGACA

CT C/TW-3 GTTT-TTCTTATCAACT**TTACGA**GAATAAGAAAGTTT**TG**T**TATGGT**CTCGAGCATTGAACGATATGTTCTCGATTAAGGCTGCTTT-TACTTGCAAGACA

CT D/14-96 GTTT-TTCTTATCAACT**TTACGA**GAATAAGAAAATTT**TG**T**TATGGT**CTCGAGCATTGAACGACATGTTCTCGATTAAGGCTGCTTT-TACTTGCAAGACA

CT E/SW3 GTTT-TTCTTATCAACT**TTACGA**GAATAAGAAAATTT**TG**T**TATGGT**CTCGAGCATTGAACGACATGTTCTCGATTAAGGCTGCTTT-TACTTGCAAGACA

CT F/SW4 GTTT-TTCTTATCAACT**TTACGA**GAATAAGAAAATTT**TG**T**TATGGT**CTCGAGCATTGAACGACATGTTCTCGATTAAGGCTGCTTT-TACTTGCAAGACA

CT G/9301 GTTT-TTCTTATCAACT**TTACGA**GAATAAGAAAATTT**TG**T**TATGGT**CTCGAGCATTGAACGACATGTTCTCGATTAAGGCTGCTTT-TACTTGCAAGACA

CT H GTTT-TTCTTATCAACT**TTACGA**GAATAAGAAAATTT**TG**T**TATGGT**CTCGAGCATTGAACGACATGTTCTCGATTAAGGCTGCTTT-TACTTGCAAGACA

CT Ia/20-97 GTTT-TTCTTATCAACT**TTACGA**GAATAAGAAAATTT**TG**T**TATGGT**CTCGAGCATTGAACGACATGTTCTCGATTAAGGCTGCTTT-TACTTGCAAGACA

CT J/31-98 GTTT-TTCTTATCAACT**TTACGA**GAATAAGAAAATTT**TG**T**TATGGT**CTCGAGCATTGAACGACATGTTCTCGATTAAGGCTGCTTT-TACTTGCAAGACA

CT K/SotonK1 GTTT-TTCTTATCAACT**TTACGA**GAATAAGAAAATTT**TG**T**TATGGT**CTCGAGCATTGAACGACATGTTCTCGATTAAGGCTGCTTT-TACTTGCAAGACA

CT L1/115 GTTT-TTCTTATCAACT**TTACGA**GAATAAGAAAATTT**TG**T**TATGGT**CTCGAGCATTGAACGACATGTTCTCGATTAAGGCTGCTTT-TACTTGCAAGACA

CT L2/434/Bu GTTT-TTCTTATCAACT**TTACGA**GAATAAGAAAATTT**TG**T**TATGGT**CTCGAGCATTGAACGACATGTTCTCGATTAAGGCTGCTTT-TACTTGCAAGACA

CT L2b/795 GTTT-TTCTTATCAACT**TTACGA**GAATAAGAAAATTT**TG**T**TATGGT**CTCGAGCATTGAACGACATGTTCTCGATTAAGGCTGCTTT-TACTTGCAAGACA

CT L2c GTTT-TTCTTATCAACT**TTACGA**GAATAAGAAAATTT**TG**T**TATGGT**CTCGAGCATTGAACGACATGTTCTCGATTAAGGCTGCTTT-TACTTGCAAGACA

CT L3/404/LN GTTT-TTCTTATCAACT**TTACGA**GAATAAGAAAATTT**TG**T**TATGGT**CTCGAGCATTGAACGACATGTTCTCGATTAAGGCTGCTTT-TACTTGCAAGACA

CAB S26-3 CTTTTTTCTTATCGTCT**TTACTA**TAATAAGAAAAGTT**TG**T**TATGTT**TTCGAATAATGAACTGTATGTTCATGCTTAAGGCTGTTTT-CACTTGCAAGACA

CAV 10DC88 CTTT-TTCTTATCGTCT**TTACTA**TAATAAGGAAAGTT**TG**T**TATGTT**TTCGAGTAGTGAACTGTATGTTCATGCTTAAGGCTGTTTT-TATT--CAAGACG

CCA GPIC GTTTTTTCTTATCGTCT**TTACTA**TAATAAGAAAAGTT**TG**T**TATGTT**TTCGATTAATGAACTGTATGTTCATGCTTAAGGCTGTTTT-CACTTGCAAGACA

CFE Fe/C-56 CTTTTTTCTTATCGTCT**TTACTA**TAATAAGAAAAGTT**TG**T**TATGTT**TTCGAATAATGAACTGTATGTTCATGCTTAAGGCTGTTTT-CACTTGCAAGACA

CG JXC CTTTATTCTTATCGTCT**TTACTA**TAATAAGAAAAGTT**TG**T**TATGTT**TTCGAGTAGTGAACTGTATGTTCATGCTTAAGGCTGTTTT-TACTTGCAAGACA

CM MoPn/Nigg GTTT-TTCTTATCAACT**TTACGA**GAATAAGAAAATTT**TG**T**TATGGT**CTCGAGCATTGAACGACATGTTCTCGATTAAGGCTGCTTT-TACTTGCAAGACA

CPE E58 ATTT-TTCTTATCGTCT**TTACTA**TAAAAAGAAAAGTT**TG**T**TATGTT**TTCGATTAATGAGCTGTGTGTTCATGCTTAAGGCTGTTTT-CACTTGCAAGACC

CPN TW-183 AATC-TTCTTATCGTCT**TTACTA**TAATAAGAAAAGTT**TG**A**TATGTT**TTCGACTAATGAGCTGTATGTTCATATTTAAGGCCGTTTTTCAATGATAAGAGC

CPS 6BC CTTTTTTCTTATCGTCT**TTACTA**TAATAAGAAAAGTT**TG**T**TATGTT**TTCGAATAATGAACTGTATGTTCATGCTTAAGGCTGTTTT-CACTTGCAAGACA

P3 -35 P3 -10

CT A/Har-13 TTCCTCAG-GCCATTAATTGC-TACAGGACATCTTGTCTGGCTTTAACTAGGACACA-**TATACA**CCAGAA----AA----AGATAGCGAG**CACAAA**GAGA

CT B/Jali20-OT TTCCTCAG-GCCATTAATTGC-TACAGGACATCTTGTCTGGCTTTAACTAGGACACA-**TATACA**CCAGAA----AA----AGATAGCGAG**CACAAA**GAGA

CT C/TW-3 TTCCTCAG-GCCATTAATTGC-TACAGGACATCTTGTCTGGCTTTAACTAGGACGCA-**TATACA**CCAGAA----AA----AGATAGCGAG**CACAAA**GAGA

CT D/14-96 TTCCTCAG-GCCATTAATTGC-TACAGGACATCTTGTCTGGCTTTAACTAGGACGCA-**TATACA**CCAGAA----AA----AGATAGCGAG**CACAAA**GAGA

CT E/SW3 TTCCTCAG-GCCATTAATTGC-TACAGGACATCTTGTCTGGCTTTAACAAGGACGCA-**TATACA**CCAGAA----AA----AGATAGCGAG**CACAAA**GAGA

CT F/SW4 TTCCTCAG-GCCATTAATTGC-TACAGGACATCTTGTCTGGCTTTAACTAGGACGCA-**TATACA**CCAGAA----AA----AGATAGCGAG**CACAAA**GAGA

CT G/9301 TTCCTCAG-GCCATTAATTGC-TACAGGACATCTTGTCTGGCTTTAACTAGGACGCA-**TATACA**CCAGAA----AA----AGATAGCGAG**CACAAA**GAGA

CT H TTCCTCAG-GCCATTAATTGC-TACAGGACATCTTGTCTGGCTTTAACTAGGACGCA-**TATACA**CCAGAA----AA----AGATAGCGAG**CACAAA**GAGA

CT Ia/20-97 TTCCTCAG-GCCATTAATTGC-TACAGGACATCTTGTCTGGCTTTAACTAGGACGCA-**TATACA**CCAGAA----AA----AGATAGCGAG**CACAAA**GAGA

CT J/31-98 TTCCTCAG-GCCATTAATTGC-TACAGGACATCTTGTCTGGCTTTAACAAGGACGCA-**TATACA**CCAGAA----AA----AGATAGCGAG**CACAAA**GAGA

CT K/SotonK1 TTCCTCAG-GCCATTAATTGC-TACAGGACATCTTGTCTGGCTTTAATAAGGACGCA-**TATACA**CCAGAA----AA----AGATAGCGAG**CACAAA**GAGA

CT L1/115 TTCCTCAG-GCCATTAATTGC-TACAGGACATCTTGTCTGGCTTTAACTAGGACGCA-**TATACA**CCAGAA----AA----AGATAGCGAG**CACAAA**GAGA

CT L2/434/Bu TTCCTCAG-GCCATTAATTGC-TACAGGACATCTTGTCTGGCTTTAACTAGGACGCA-**TATACA**CCAGAA----AA----AGATAGCGAG**CACAAA**GAGA

CT L2b/795 TTCCTCAG-GCCATTAATTGC-TACAGGACATCTTGTCTGGCTTTAACTAGGACGCA-**TATACA**CCAGAA----AA----AGATAGCGAG**CACAAA**GAGA

CT L2c TTCCTCAG-GCCATTAATTGC-TACAGGACATCTTGTCTGGCTTTAACTAGGACGCA-**TATACA**CCAGAA----AA----AGATAGCGAG**CACAAA**GAGA

CT L3/404/LN TTCCTCAG-GCCATTAATTGC-TACAGGACATCTTGTCTGGCTTTAACTAGGACGCA-**TATACA**CCAGAA----AA----AGATAGCGAG**CACAAA**GAGA

CAB S26-3 CTCCTCAAAGCCATTAATTGCCTACAGGATATCTTGTCTGGCTTTAACTTGGACGTG-**TATACA**CCAGAAGAGCAAATTAGAATAGCGAG**CACAAA**AAGA

CAV 10DC88 TTCCTCAAAGCCATTAATTGCCTACAGGATATCTTGTCTGGCTTTAACTTGGACATG-**TATACA**CCAAAAGAGCAA----AAATAGCGAG**CACAAA**AAGA

CCA GPIC CTCCTCAAAGCCATTAATTGCCTACAGGATATCTTGTCTGGCTTTAACTTGGACGTG-**TATACA**CCAGAAGAGCAATTTAGAATAGCGAG**CACAAA**AAGA

CFE Fe/C-56 CTCCTCAAAGCCATTAATTGCCTACAGGATATCTTGTCTGGCTTTAACTTGGACGTG-**TATACA**CCAGAAGAGCAAATTAGAATAGCGAG**CACAAA**AAGA

CGA JXC TTCCTCAAAGCCATTAATTGCCTACAGGATATCTTGTCTGGCTTTAACTTGGACATG-**TATACA**CCAAAAGAGAAA----AAATAGCGAG**CACAAA**AAGA

CM MoPn/Nigg CTCCTCAG-AGCCATAATTGC-TACAGGACATCTTGTCTGGCTTTAACTAGGACACA-**TATACA**CCAGAA----AA----AGATAGCGGG**CACAAA**GAGA

CPE E58 CTCCTAACAGTCATAAAATGCCTACAGGA-ATCTTGTCTGGCTTTAACTTGAACGTAA**TATACA**CCAGAAAA-CAAGAAAATATAGCGAG**CACAAA**AAGA

CPN TW-183 TTCCTAA--------ATTTGCCTGCAGGATATCTTGTCTGGCTTTAATTTGGACGTC-**GTGTCG**CCAAAATA-TGAGTAA---TAGCGAG**CACATA**AATA

CPS 6BC CTCCTCAAAGCCATTAATTGCCTACAGGATATCTTGTCTGGCTTTAACTTGGACGTG-**TATACA**CCAGAAGAGCAAATTAGAATAGCGAG**CACAAA**AAGA

P1 -35 P1 -10

CT A/Har-13 GCTAATTATACAAT--------TTAGAGGTAAGAATGAAAAAACTCTTGAAATCGGTATTAGTATTTGCCGCTT

CT B/Jali20-OT GCTAATTATACAAT--------TTAGAGGTAAGAATGAAAAAACTCTTGAAATCGGTATTAGTATTTGCCGCTT

CT C/TW-3 GCTAATTATACAATC-------TTAGAGGTAAGAATGAAAAAACTCTTGAAATCGGTATTAGTATTTGCCGCTT

CT D/14-96 GCTAATTATACAAT--------TTAGAGGTAAGAATGAAAAAACTCTTGAAATCGGTATTAGTATTTGCCGCTT

CT E/SW3 GCTAATTATACAATC-------TTAGAGGTAAGAATGAAAAAACTCTTGAAATCGGTATTAGTATTTGCCGCTT

CT F/SW4 GCTAATTATACAAT--------TTAGAGGTAAGAATGAAAAAACTCTTGAAATCGGTATTAGTATTTGCCGCTT

CT G/9301 GCTAATTATACAAT--------TTAGAGGTAAGAATGAAAAAACTCTTGAAATCGGTATTAGTATTTGCCGCTT

CT H GCTAATTATACAAT--------TTAGAGGTAAGAATGAAAAAACTCTTGAAATCGGTATTAGTATTTGCCGCTT

CT Ia/20-97 GCTAATTATACAATC-------TTAGAGGTAAGAATGAAAAAACTCTTGAAATCGGTATTAGTATTTGCCGCTT

CT J/31-98 GCTAATTATACAATC-------TTAGAGGTAAGAATGAAAAAACTCTTGAAATCGGTATTAGTATTTGCCGCTT

CT K/SotonK1 GCTAATTATACAAT--------TTAGAGGTAAGAATGAAAAAACTCTTGAAATCGGTATTAGTATTTGCCGCTT

CT L1/115 GCTAATTATACAAT--------TTAGAGGTAAGAATGAAAAAACTCTTGAAATCGGTATTAGTGTTTGCCGCTT

CT L2/434/Bu GCTAATTATACAAT--------TTAGAGGTAAGAATGAAAAAACTCTTGAAATCGGTATTAGTGTTTGCCGCTT

CT L2b/795 GCTAATTATACAAT--------TTAGAGGTAAGAATGAAAAAACTCTTGAAATCGGTATTAGTGTTTGCCGCTT

CT L2c GCTAATTATACAAT--------TTAGAGGTAAGAATGAAAAAACTCTTGAAATCGGTATTAGTGTTTGCCGCTT

CT L3/404/LN GCTAATTATACAAT--------TTAGAGGTAAGAATGAAAAAACTCTTGAAATCGGTATTAGTGTTTGCCGCTT

CAB S26-3 AA-AGATAC-TAAGCATAATCTTTAGAGGTGAGTATGAAAAAACTCTTGAAATCGGCATTATTGTTTGCCGCTA

CAV 10DC88 AAAAGATAC-TAAGCATAATCTTTAGAGGTGAGTATGAAAAAACTCTTGAAATCGGCATTATTGGTTGCCGTAG

CCA GPIC AA-AGATAC-TAAGCATAATCTTTAGAGGTGAGTATGAAAAAACTCTTGAAATCGGCATTATTGTTTGCCACTA

CFE Fe/C-56 AAAGATACTATGCAT--AATCTTTAGAGGTGAGTATGAAAAAACTCTTAAAATCGGCATTATTATTTGCCGCTG

CGA JXC AAAAGATACATAAGCATAATCTTTAGAGGTGAGTATGAAAAAACTCTTGAAATCGGCATTGTTGTTTGCCGTAG

CM MoPn/Nigg GCTAATTATACAATCTTA----TTAGAGGTAAGAATGAAAAAACTCTTGAAATCGGTATTAGCATTTGCCGTTT

CPE E58 -------AT-TAAGCATAATCTT-AGAGGTGAGTATGAAAAAACTCTTAAAATCGGCGTTTTTATCCGCCGCAT

CPN TW-183 AA-AGATAC-TAAGCATAATCTTTAGAGGTGAGTATGAAAAAACTCTTAAAGTCGGCGTTATTATCCGCCGCAT

CPS 6BC AA-AGATAC-TAAGCATAATCTTTAGAGGTGAGTATGAAAAAACTCTTGAAATCGGCATTATTGTTTGCCGCTA

Start codon

Note:

a. Strain abbreviation: CT: *Chlamydia trachomatis*; CAB: *Chlamydia abortus*; CAV: *Chlamydia avium*; CCA: *Chlamydia caviae*; CFE: *Chlamydia felis*; CGA: *Chlamydia gallinacea*; CM: *Chlamydia muridarum*; CPE: *Chlamydia pecorum*; CPN: *Chlamydia pneumoniae*; CPS: *Chlamydia psittaci*;

b. DNA sequence alignments were carried out using ClustalW[^1^](#_ENREF_1). The putative -35 and -10 hexamers, and TGn motif were indicated as bolded red font. The start codon of *ompA* coding region is indicated as green font.

**Table S2** Plasmids used in the present study

| Plasmids | Description | Source or reference |
| --- | --- | --- |
| pGFP::SW2 | A *C. trachomatis* shuttle plasmid containing P*_nm_*::GFP | [^2^](#_ENREF_2) |
| pBOMB4-tet-mCherry  (pBOMBm) | A *C. trachomatis* shuttle plasmid containing P*_tet_*::mCherry and P*_nm_*::GFP | [^3^](#_ENREF_3) |
| pPvGFP::SW2 | A P*_euo_* ::GFP reporter vector derived from pGFP::SW2 | This study |
| pPLGFP::SW2 | Promoter-less control vector | This study |
| pBOMB-P*_euo_* | A P*_euo_* ::GFP reporter vector derived from pBOMB4-tet-mCherry | This study |
| pP3GFP::SW2 | A P3::GFP reporter vector derived from pPvGFP::SW2 | This study |
| pBOMB-P3 | A P3::GFP reporter vector derived from pBOMB-P*_euo_* | This study |
| pBOMB-PL | Promoter-less control vector | This study |
| pP*_flic_* | Vector for *in vitro* transcription assay | [^4^](#_ENREF_4) |
| pP3WT-P*_fliC_* | Vector derived from pP*_flic_* with inserted P3 for *in vitro* transcriptional analysis | This study |
| pP3m10-P*_fliC_* | Vector derived from pP*_flic_* with mutated P3 in -10 hexamer | This study |
| pP3mTG-P*_fliC_* | Vector derived from pP*_flic_* with mutated P3 in -10 extend element | This study |
| pP3m35-P*_fliC_* | Vector derived from pP*_flic_* with mutated P3 in -35 hexamer | This study |
| pFW11 | *E. coli* plasmid used for conjugation transferring target sequence onto episome in host bacteria by homologous recombination | [^5^](#_ENREF_5) |
| pFWP21 | pFW11-derivative vector containing *ompA* promoter region covering P2 and P1 in the transfer cassette | This study |
| pFWP2 | pFW11-derivative vector containing *ompA* promoter region covering only P2 in the transfer cassette | This study |
| pFWP1 | pFW11-derivative vector containing *ompA* promoter region covering only P1 in the transfer cassette | This study |
| pUC19 | Cloning vector for sequencing | Promega, |

**Table S3. Primers used in the present study**

| **Primers** | **Sequence** | **purpose** |
| --- | --- | --- |
| SW2-Pv1 | TGGGAAAATGTTACCTTCTCTTTTGCCGGCAGCAAACTAATTTTTAATTTGAGGATTTTTGAGATGAGTAAAGGAGAAGCACTTTTCACTGGAGTTGT | pPvGFP::SW2 |
| SW2_Pv2 | TGGGAAAATGTTACCTTCTCTTTTGCCGGCAGCAAACTAATTTTTAATTTGAGGATTTTTGAGATGAGTAAAGGAGAAGCACTTTTCACTGGAGTTGT |
| *ompA* proF | ATGAATTCAACAATCAACATCTTGCCAAC | pFWP21 |
| *ompA* P1R | ACTCGACTAATTAGCTCTCTTTGTGCTCGCTATCTT |
| *ompA*P2EcoSalU _U | AATTCGCAAAAACCAAGAACATAAAAACATAAAAAGA**TATACA**AAAATGGCTCTCTGCTT**TATACA**TAAATCAGGAG | pFWP2 |
| *ompA*P2 EcoSalU _L | TGCACTCCTGATTTAGCAATAAAGCAGAGAGCCATTTTTGTATATCTTTTTATGTTTTTATGTTCTTGGTTTTTGCG |
| *ompA*P1EcoSal_U | AATTCTTGTCTGGCTTTAACTAGGACGCA**TATACA**CCAGAAAAAGATAGCGAG**CACAAA**GAGAGCTAATTAG | pFWP1 |
| *ompA*P1EcoSal_L | tcgacTAATTAGCTCTCTTTGTGCTCGCTATCTTTTTCTGGCGGCACTGCGTCCTAGTTAAAGCCAGACAAG |
| *ompA*P3_U | CTAGAACGGTTTTTCTTATCAACT**TTACGA**GAATAAGAAAATTTTGT**TATGGT**CTCGACCATT | pGFPP3::SW2  pP3WT-P*_fliC_* |
| *ompA*P3_L | AATGGTCGAGACCATAACAAAATTTTCTTATTCTCGTAAAGTTGATAAGAAAAACCGTT |
| s70priF | ATCTCCATGGAAACGCCGATC | pLN-σ^66R24^ |
| sig7066Rg24CUp | CGTATGTCCATCGGTGAAGCGAAAGCCCAGGAA |
| Sig7066Rg24BLo | CACCATCTCTTTTTTGGCTTCCTGGGCTTTCGC |
| S66prRBam | GACTGGATCCCTAATTTTTATAACTTTTAATCTTACCCGAACCAATTT |
| 5’*ompA*RACEouter | CTCAGCTGCGTTACAGAGAACGTT | 5’RACE assay |
| 5’*ompA*RACEinner | CGCCTGCAGG**TATACA**ACTCAACAACAGATTGA, |
| 5’*ompA*RACEspecific | CCTGCTGAACCAAGCCTTATGATC |
| *ompA*P3m-35_U | CTAGAACGGTTTTTCTTATCAACTGGATCCGAATAAGAAAATTTTGT**TATGGT**CTCGACCATT | pP3m35-P*_fliC_* |
| *ompA*P3m-35_L | AATGGTCGAGACCATAACAAAATTTTCTTATTCGGATCCAGTTGATAAGAAAAACCGTT |
| *ompA*P3mTG_U | CTAGAACGGTTTTTCTTATCAACT**TTACGA**GAATAAGAAAATTTCAT**TATGGT**CTCGACCATT | pP3mTG-P*_fliC_* |
| *ompA*P3mTG_L | AATGGTCGAGACCATAATGAAATTTTCTTATTCTCGTAAAGTTGATAAGAAAAACCGTT |
| *ompA*P3m-10_U | CTAGAACGGTTTTTCTTATCAACT**TTACGA**GAATAAGAAAATTTTGTGCATGCCTCGACCATT | pP3m10-P*_fliC_* |
| *ompA*P3m-10_L | AATGGTCGAGGCATG**CACAAA**ATTTTCTTATTCTCGTAAAGTTGATAAGAAAAACCGTT |
| QP2*ompA*F | GGCGCTTAAGGGCTTCTTCC | RT-qPCR |
| QP2*ompA*R | AATGGCCTGAGGAATGTCTTGC |
| QP2*ompA*/3F | CGACATGTTCTCGATTAAGGCTGC | RT-qPCR |
| QP2*ompA*/3R | GCGGCACTGCGTCCTAGTTAAA |
| Q16SF | CGCCAACACTGGGACTGAGA | qPCR |
| Q16SR | GGCGTCGCTTCGTCAGACTT |

References:

1 Goujon, M., McWilliam, H., Li, W., Valentin, F., Squizzato, S., Paern, J., Lopez, R A new bioinformatics analysis tools framework at EMBL-EBI. *Nucleic acids research* **Suppl: 38** W695-699, doi:doi:10.1093/nar/gkq313 (2010).

2 Wang, Y., Kahane, S., Cutcliffe, L. T., Skilton, R. J., Lambden, P. R., Clarke, I. N. Development of a transformation system for *Chlamydia trachomatis*: restoration of glycogen biosynthesis by acquisition of a plasmid shuttle vector. *PLoS Pathog* **7**, e1002258, doi:10.1371/journal.ppat.1002258 (2011).

3 Bauler, L. D. & Hackstadt, T. Expression and targeting of secreted proteins from *Chlamydia trachomatis*. *J Bacteriol* **196**, 1325-1334, doi:10.1128/JB.01290-13 (2014).

4 Shen, L., Li, M., Zhang, Y. X. *Chlamydia trachomatis* sigma28 recognizes the *fliC* promoter of *Escherichia coli* and responds to heat shock in chlamydiae. *Microbiology* **150**, 205-215 (2004).

5 Whipple, F. W. Genetic analysis of prokaryotic and eukaryotic DNA-binding proteins in Escherichia coli. *Nucleic acids research* **26**, 3700-3706 (1998).
